# Pulmonary Metastases of Mixed Adenoid Cystic and Squamous Cell Carcinoma of the Uterine Cervix: A Report of a Rare Case

**DOI:** 10.7759/cureus.77822

**Published:** 2025-01-22

**Authors:** Meriem Rhazari, Afaf Thouil, Imane Kamaoui, Hatim Kouismi

**Affiliations:** 1 Department of Respiratory Diseases, Research and Medical Sciences Laboratory, Faculty of Medicine and Pharmacy of Oujda, Mohammed VI University Hospital, Mohammed First University, Oujda, MAR; 2 Department of Radiology, Faculty of Medicine, Mohammed VI University Hospital, Mohammed First University, Oujda, MAR

**Keywords:** adenoid cystic carcinoma (acc), epidermoid carcinoma, lung cancer, mixed carcinoma, uterine cervix

## Abstract

Cystic adenoid carcinoma (CAK) is a rare malignant tumor primarily affecting the salivary glands, but it can also involve other sites, including the uterine cervix. This tumor is associated with a high potential for local recurrence and pulmonary metastasis. We present the case of a 73-year-old diabetic woman with a history of cervical adenocarcinoma treated with radiochemotherapy and total hysterectomy. She was hospitalized for acute dyspnea, and imaging revealed pulmonary lesions, which were confirmed by biopsy as pulmonary metastases of CAK. A review of the hysterectomy specimen revealed a mixed carcinoma with both adenoid cystic and squamous cell components. The patient was started on palliative chemotherapy. The diagnosis and management of mixed carcinomas of the uterine cervix, including CAK, remain complex and require a multimodal approach.

## Introduction

Cystic adenoid carcinoma (CAC) is a rare malignant tumor [1]. Although it primarily arises in the salivary glands, it can also affect other glandular tissues, including the nasal passages, tracheobronchial tree, and, less commonly, gynecological sites such as the uterine cervix. In these rare locations, CAC is often associated with distinct clinical and pathological features [1].

CAC is a locally aggressive tumor with a high propensity for local recurrence and distant metastasis [2]. Its diagnosis is challenging due to its subtle clinical presentation and slow progression. The prognosis of patients with CAC depends on several factors, including tumor location, metastatic involvement, and response to therapy [2].

In the uterine cervix, CAC has been associated with other conditions, such as cervical intraepithelial neoplasia, invasive squamous cell carcinoma, adenocarcinoma, and sarcoma. However, the relationship between CAC and these tumors remains poorly understood and underexplored [3].

The aim of this article is to present a rare clinical case of a patient initially diagnosed with adenocarcinoma of the uterine cervix, which was subsequently identified as a mixed carcinoma consisting of a CAC component and a squamous cell carcinoma component, accompanied by pulmonary metastases. This report will address its diagnostic challenges, histopathological characteristics, therapeutic strategies, and management.

## Case presentation

A 73-year-old female patient, with a history of diabetes managed with insulin therapy and oral antidiabetic medications, was treated for Pott's disease 14 years ago and has been followed for adenocarcinoma of the uterine cervix, for which she underwent radiochemotherapy and total hysterectomy two years ago. She was hospitalized due to the acute worsening of chronic dyspnea, occurring at rest and without other associated symptoms. The condition developed in the context of overall health deterioration. On clinical examination, the patient exhibited oxygen desaturation to 79% on ambient air, which improved to 93% with four liters per minute of oxygen therapy, without signs of respiratory distress.

**Figure 1:**
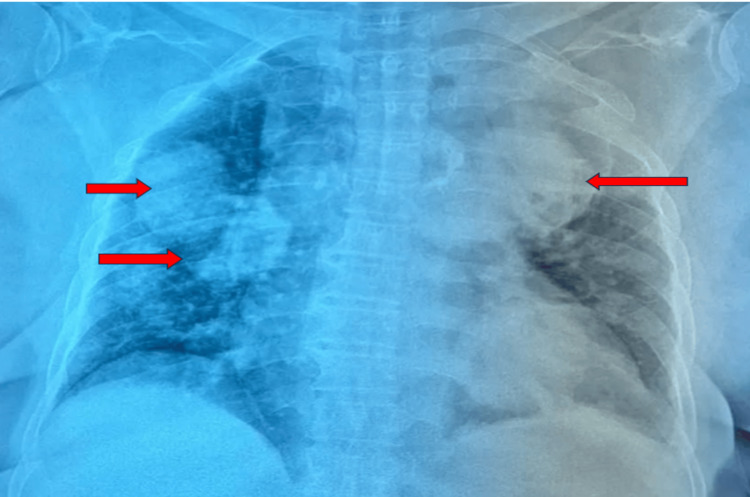
The frontal chest X-ray showed multiple rounded opacities with a watery tone and a clear boundary located in the two pulmonary hemichambers, giving a balloon-like appearance. Red arrow: Highlights the multiple rounded opacities with a watery tone and sharp edges located in the two pulmonary hemichannels.

A frontal chest X-ray revealed multiple rounded opacities with a water-density appearance and well-defined borders, distributed bilaterally in the pulmonary fields, giving a characteristic "balloon-like" appearance (Figure 1).

**Figure 2:**
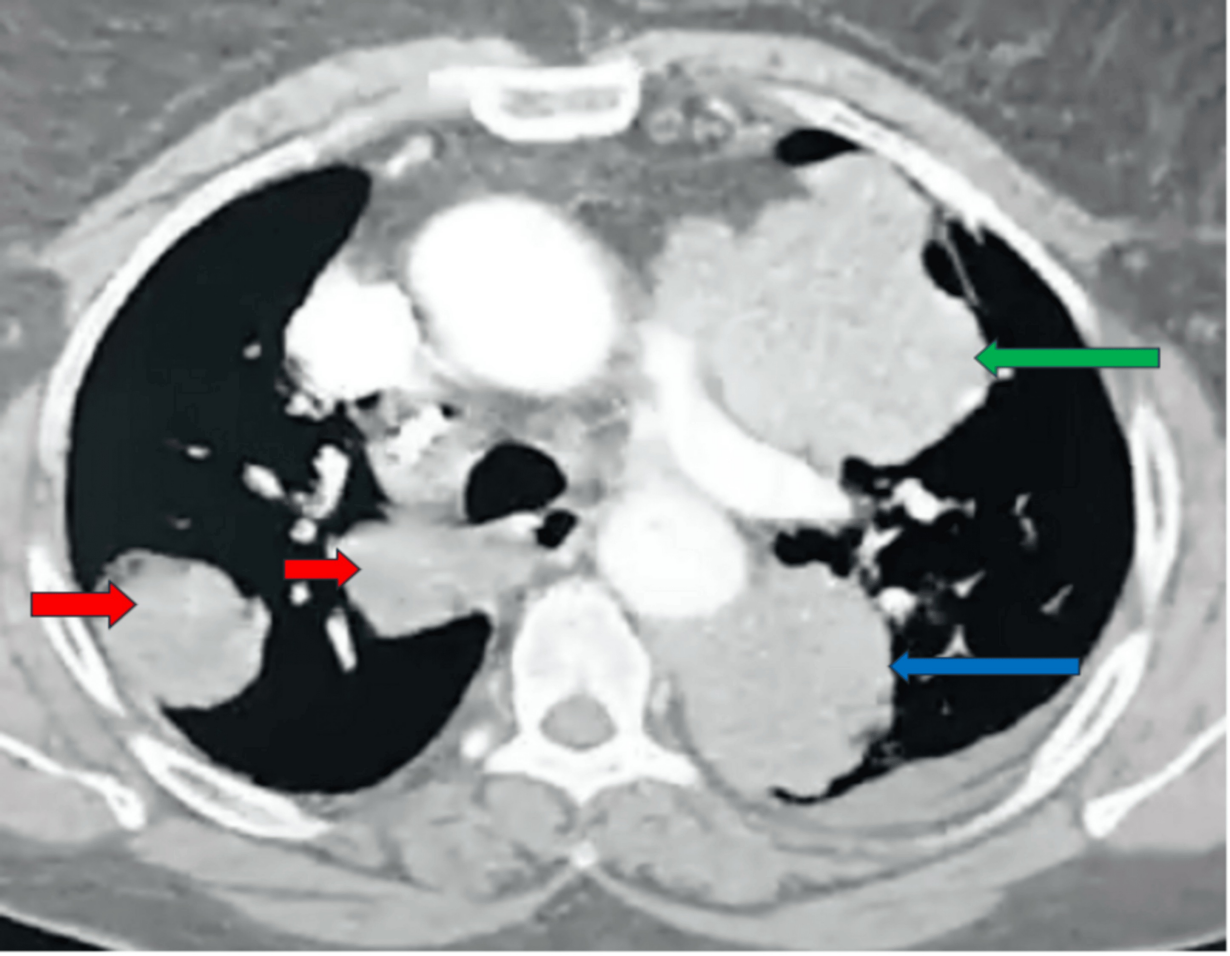
Thorax CT scan with mediastinal window demonstrating two voluminous lesion processes, one posteroapical on the left and the other anteroapical on the left, with the presence of multiple nodular and macronodular formations on the right, appearing secondary in nature. Blue arrow: Indicates a voluminous lesion process in the left posteroapical position, measuring 74 x 69 mm, with pleural involvement in its external part. Green arrow: Highlights a second, similar lesion process, located in the left anteroapical position, measuring 50 x 40 mm, grazing the hemi-circumference of the pulmonary artery trunk and presenting an external parenchymal component. Red arrows: Point to multiple nodular and macronodular formations on the right, the largest measuring 38 x 37 mm.

A thoracic angio-CT scan revealed a large left posteroapical lesion measuring 74 x 69 mm, with pleural involvement at its outer margin. This lesion abuts the hemicircumference of the descending aorta and the ipsilateral pulmonary artery. A second similar lesion was identified in the left anteroapical region, measuring 50 x 40 mm, in close proximity to the hemi-circumference of the pulmonary artery trunk, with a parenchymal external component. Additionally, multiple bilateral (right and left) nodular and macronodular formations were observed, the largest of which measured 38 x 37 mm on the right and 49 x 42 mm on the left (Figure 2).

The patient underwent bronchoscopy, which showed no particular findings, and the search for neoplastic cells in the bronchial aspirate fluid was negative.

**Figure 3:**
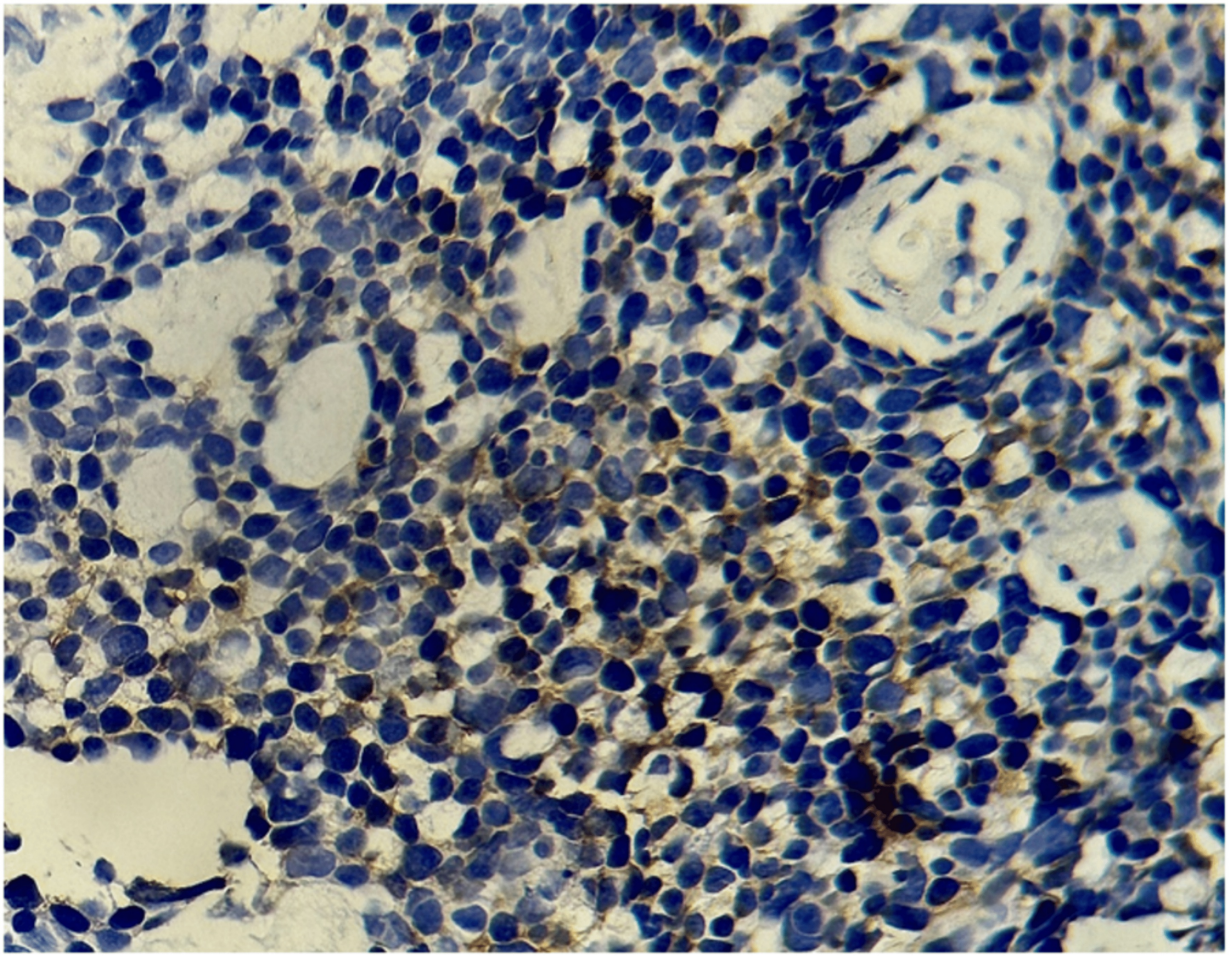
Focal, weakly positive staining of tumor cells with the anti-CD117 antibody.

At this stage, it was necessary to perform a CT-guided biopsy of the lung lesion. The histological and immunohistochemical analysis was consistent with CAC (Figure 3).

**Figure 4:**
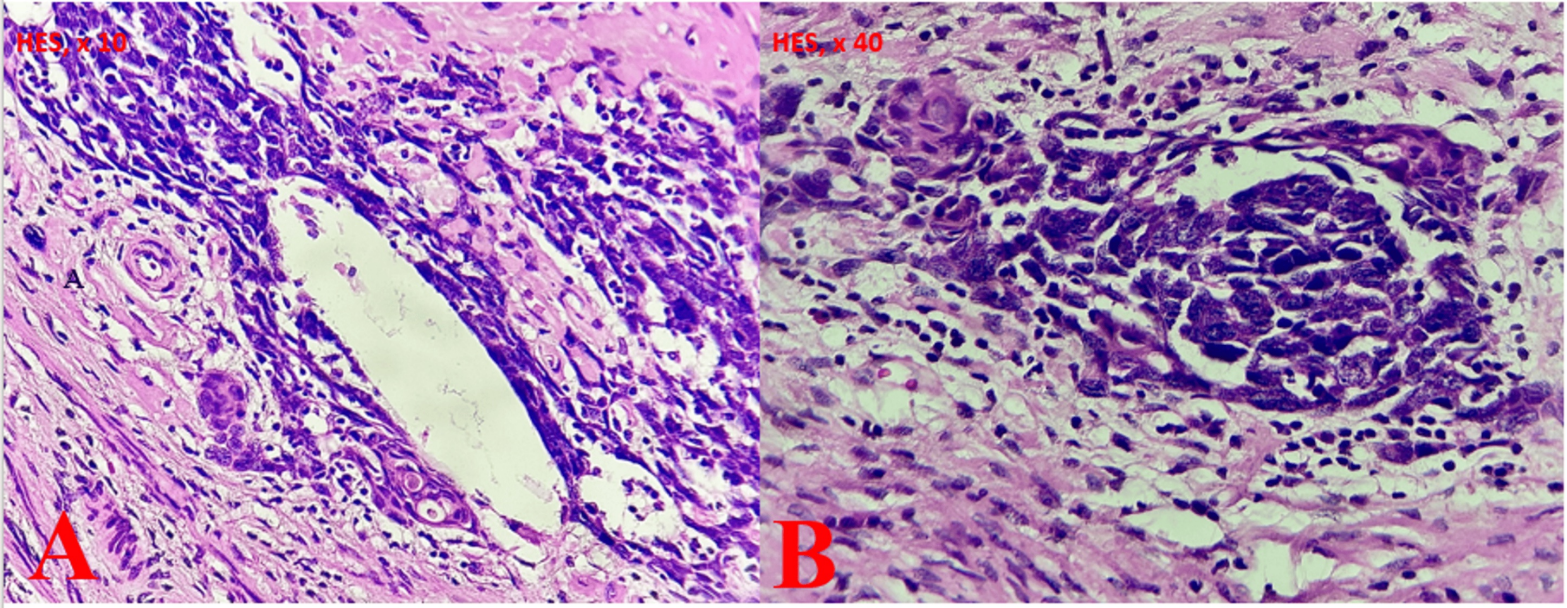
Histological images showing an association between squamous cell carcinoma (A) and adenoid cystic carcinoma (B).

In light of the anatomopathological findings from the lung biopsy, we considered a secondary endometrial origin, which prompted a re-evaluation of the hysterectomy specimen. The results were consistent with a mixed squamous cell carcinoma and CAC (Figure 4). The course of action was to initiate palliative chemotherapy, consisting of three cycles, followed by reassessment.

## Discussion

Mixed carcinomas of the uterine cervix are a rare and aggressive form of neoplasia, characterized by the coexistence of two distinct components: a CAK and a squamous cell component. This combination makes the diagnosis and management of these tumors particularly challenging. Adenoid cystic carcinoma (ACC), a rare but well-established histological variant, is known for its cribriform glandular structure and slow progression, although it can also metastasize to distant sites, notably the lungs and liver [4]. These tumors are often misdiagnosed or confused with more common cervical carcinomas. For example, a mixed tumor predominantly composed of glandular cells may initially be misinterpreted as an adenocarcinoma, and it is only after extensive immunohistochemical analysis that its mixed nature is identified [5], as in the case of our patient, who was initially diagnosed with cervical adenocarcinoma.

The first case of ACC of the uterine cervix, initially described as a cylindroma, was reported in 1949. ACC accounts for less than 1% of all cervical carcinomas, making it an extremely rare malignancy [6]. It is characterized by slow growth and hematogenous dissemination, primarily to the lungs [7]. In some cases, the exclusive presence of CAK-type metastases in the lungs, in the absence of a squamous cell component, suggests selective dissemination or tumor plasticity, potentially linked to the clonal evolution of tumor cells or to microenvironmental factors favoring the proliferation of the CAK component [8].

Pulmonary involvement, although rare, is characterized by a prolonged asymptomatic course, with respiratory symptoms (persistent cough, dyspnea, or chest pain) only appearing at an advanced stage. Histologically, it presents a tubulo-cribriform architecture with mucus-filled cystic spaces, which differentiates it from classical lung adenocarcinomas [9,10].

P63 is a useful marker that shows strong, diffuse nuclear staining in squamous cell carcinoma, but a peripheral or compartmentalized distribution in ACC. Squamous cell carcinoma is negative for CK7 and CD117, while the luminal component of CAK is positive for these markers [3]. The diagnosis of CAK lung metastases is based on clinical, radiological, and histopathological analyses. Thoracic imaging, which may reveal nodules or parenchymal masses, is crucial for confirming the diagnosis. Differentiation from other tumors, such as adenocarcinomas or metastases of different origins, remains a significant challenge [2,3,8].

The treatment of lung metastases from mixed carcinoma of the uterine cervix follows a multimodal approach, combining surgery, radiotherapy, and chemotherapy. In cases of localized, resectable metastases, surgical resection, such as lobectomy, can improve long-term survival. Conformal radiotherapy is effective for recurrent or locally advanced disease, while chemotherapy (carboplatin, paclitaxel) is indicated for distant metastases [6,7], as in our patient's case, where treatment consisted of palliative chemotherapy.

Despite the slow progression of CAK, the presence of pulmonary metastases, particularly when associated with squamous cell carcinoma, is an unfavorable prognostic factor [8]. Innovative treatments, such as immunotherapy or targeted therapies, though not extensively studied in this context, could offer new therapeutic avenues.

## Conclusions

Mixed cervical carcinomas, although rare, present major diagnostic and therapeutic challenges due to the coexistence of adenoid cystic and epidermoid components. Diagnosis relies on precise histopathological and immunohistochemical analyses. Pulmonary metastases can complicate the progression, requiring a multimodal approach for management.
